# When Less Confidence Reaps More AI Benefits: A Compensatory Pattern Through Future Work Self-Salience

**DOI:** 10.3390/bs16091585

**Published:** 2026-09-07

**Authors:** Xiaobo Dong, Yanlong Zhang, Qi Li, Zhiyong Han

**Affiliations:** 1 China Cooperative Research Institute, Anhui University of Finance and Economics, Bengbu 233030, China; 2 School of Business Administration, Anhui University of Finance and Economics, Bengbu 233030, China; 3202400113@aufe.edu.cn (Y.Z.);

**Keywords:** employee–AI collaboration, employee innovation behavior, social cognitive theory, core self-evaluation, future work self-salience

## Abstract

**Introduction:** In the context of AI becoming deeply embedded in professional environments, understanding how EAC drives employee innovative behavior (EIB) has become a critical issue in organizational psychology. Drawing on social cognitive theory (SCT) and complementarity theory, this study proposes an “Environment-Cognition-Behavior” framework to elucidate how employee–AI collaboration (EAC) drives EIB, specifically by assessing the mediating effect of future work self-salience (FWS) and the moderating effect of core self-evaluation (CSE). **Methods:** A three-wave time-lagged questionnaire survey was employed, and 457 valid employee responses were collected via the Credamo platform. The moderated mediation model was tested using regression-based path analysis and bootstrapping procedures. **Results:** EAC significantly and positively predicted EIB. FWS significantly mediated this relationship. CSE moderated the effect of EAC on FWS, yielding a significant moderated mediation effect. Notably, among employees with low CSE, the indirect pathway from EAC to EIB via FWS was stronger. **Discussion:** These findings extend SCT by identifying a pattern of person–environment interaction that is consistent with a compensatory interpretation in human–AI collaboration contexts. They also clarify the cognitive mechanism and individual-level boundary condition underlying AI-enabled innovation and provide practical implications for differentiated AI management strategies.

## 1. Introduction

Artificial intelligence (AI) is fundamentally reshaping how work is organized and executed, with organizations increasingly deploying AI systems not merely as automation tools but as collaborative agents that interact with employees in task execution and decision-making (Qin & Zhao, 2025; J. Y. Chen et al., 2025; Jiang et al., 2024). This shift raises a critical question for organizational behavior research: can collaboration with AI stimulate employees’ creativity and innovative potential (Luo & Cai, 2026; Gao et al., 2026)? Unlike traditional technologies, AI exhibits adaptive, learning, and generative capabilities that fundamentally alter the nature of human–technology interaction (Seeber et al., 2020; Zheng et al., 2025). Employee–AI collaboration (EAC)—defined as the process through which employees interact with AI tools, systems, or algorithms to jointly accomplish work tasks—thus represents a qualitatively new work context that may have profound implications for employee cognition and behavior (Y. W. Liu & Huang, 2026; Luo & Cai, 2026). However, despite growing recognition of EAC’s performance-enhancing potential, the psychological mechanisms through which EAC translates into employee innovation behavior (EIB), and the boundary conditions that shape this translation, remain insufficiently understood.

Existing studies on the influence of AI on workplace behavior have yielded substantial insights. Research on the negative effects of AI has primarily examined employee anxiety and job insecurity (Kaya et al., 2024; He et al., 2025). In contrast, research on its positive impacts has emphasized performance improvement (He et al., 2024), as well as its varying effects on work efficiency, learning ability, and skill development (Kong et al., 2023; Ma & Zhou, 2025; Xie & Wang, 2025; M. X. Sun et al., 2025). In the domain of EIB, the existing literature has extensively examined the influences of organizational support, leadership style, team characteristics, as well as individual motivation and self-efficacy (Han & Ni, 2025; Lin et al., 2024). Some studies have begun to address the link between AI technology usage and innovation, but they often remain at the level of technology adoption or tool utilization, without deeply analyzing the psychological and cognitive processes generated when employees interact and collaborate with AI. Although prior studies have provided valuable insights, several research gaps still exist in the literature on EAC and innovation. Most researchers regard AI as an external tool, ignoring how EAC, as an emerging work context, promotes innovation by shaping employees’ internal cognitive processes. Therefore, we integrate SCT and complementarity theory to address this issue.

SCT posits that human behavior arises from the ongoing interplay among personal, behavioral, and environmental determinants. Individuals learn through observation, modeling, and self-regulation, and cognitive processes play a central role in shaping behavior (Bandura, 1986, 1991). Within the framework of SCT, when employees engage in ongoing EAC, receive feedback and suggestions from AI, and adapt to new work paradigms, they constantly evaluate their capabilities and future development trajectories, thereby gradually forming greater future work self-salience (FWS). FWS refers to the clear mental representation employees construct regarding their future occupational roles (Strauss et al., 2012; Voigt & Strauss, 2024). Increased FWS enhances employees’ sense of control and reduces cognitive load (C. X. Sun et al., 2025), enabling them to reallocate cognitive resources to in-depth reflection and career planning (Zhang et al., 2017; Chang et al., 2026), ultimately improving their perceived competence and environmental predictability (Parker & Grote, 2022). Research has shown that FWS serves as a critical motivational driver of proactive behaviors such as innovation (Yang et al., 2023; Guan et al., 2017). It provides clear directional guidance and stimulates discrepancy-reduction motivation (Oh, 2020), prompting employees to narrow the gap between their current and ideal selves through EIB. Accordingly, EAC indirectly fosters innovation behavior by enhancing FWS, and FWS acts as a key cognitive bridge linking EAC to EIB.

SCT proposes that person–environment interaction shapes individuals’ processing of environmental information and self-regulation processes (Bandura, 1986). Accordingly, we introduce CSE as a moderating variable. CSE is a broad personality construct encompassing self-esteem, locus of control, and emotional stability (Judge & Kammeyer-Mueller, 2011; Srivastava et al., 2010), which significantly influences how individuals perceive, interpret, and respond to external information and situations. This raises an important question: in a high-resource context such as EAC, will the coexistence of two high-level factors—strong collaboration with AI and high core self-evaluations—create interference or conflict, such that the effect of one or both becomes nonsignificant or even disappears? While existing research predominantly focuses on the positive effects of CSE (Y. E. Chen & Lai, 2025; N. Liu et al., 2026), this study further incorporates complementarity theory (Kiesler, 1983) to advance a more dialectical perspective. The core premise of complementarity theory is that individuals tend to seek complementary fit to maintain balance between their personal attributes and external conditions. Specifically, employees with high CSE typically possess a more stable and positive self-concept (Y. E. Chen & Lai, 2025; N. Liu et al., 2026). They may rely less on external signals derived from EAC to reconstruct their understanding of their future work lives. Thus, the enhancing effect of EAC on FWS will be relatively weak or even nonsignificant for them. In contrast, employees with low CSE may be more inclined to seek guidance and feedback from EAC to construct or revise their future work selves. As a result, the positive impact of EAC on FWS will be stronger for these individuals. Therefore, this study investigates how core self-evaluation (CSE) moderates the relationship between EAC and FWS, proposing that CSE shapes the intensity of this association, thereby revealing the interindividual differential pathways through which EAC influences innovation.

This study makes three interconnected theoretical contributions. First, we extend SCT by specifying a compensatory—rather than synergistic—mode of person–environment interaction in human–AI collaboration. Second, we identify FWS as a cognitive mediator, revealing a pathway from EAC to innovation that is conceptually distinct from prior motivational accounts. Third, we establish CSE as a boundary condition that moderates the cognitive benefits of EAC, suggesting that external AI resources may compensate for—rather than merely amplify—internal psychological resources, depending on individual differences.

## 2. Theoretical Basis and Research Hypotheses

### 2.1. SCT and Complementarity Theory

This theoretical model integrates SCT (Bandura, 1986) and complementarity theory (Kiesler, 1983). SCT provides the overarching Environment–Cognition–Behavior framework, specifying that external work contexts shape individual behavior through cognitive restructuring processes. Complementarity theory, originally developed to describe interpersonal interaction patterns (Kiesler, 1983), provides a useful heuristic for understanding person–environment resource dynamics. Its core logic that individuals adjust their responses to compensate for perceived deficits in the other party can be conceptually translated to the resource domain: when personal resources are low, individuals become more responsive to environmental resources, whereas when personal resources are high, external inputs yield diminishing marginal returns. Although Kiesler’s framework did not directly address human–technology interaction, its underlying principle has been extended to resource-based person–environment exchanges in organizational research (Edwards & Rothbard, 1999; Hobfoll, 1989), and is consistent with the broader job demands–resources logic that external resources matter most when personal resources are deficient (Bakker & Demerouti, 2017; Ten Brummelhuis & Bakker, 2012). In the present study, we apply this logic to the human–AI collaboration context by treating EAC as an external resource and CSE as an internal personal resource, proposing that the cognitive benefit of EAC on FWS will be more pronounced when CSE is low—a pattern we term resource complementarity in the person–environment interaction.

### 2.2. EAC and EIB

SCT holds that individual behavior results from the dynamic interaction between the external environment and internal cognitive factors (Bandura, 1986). When employees work in an environment characterized by collaboration with AI, their behavior is inevitably influenced by this context. Kong et al. (2023) noted that EAC can enhance employee well-being and productivity. Ma and Zhou (2025) found that EAC promotes employees’ green innovation behavior at the organizational level, and the ultimate foundation of corporate innovation lies in employee innovation. In a review of EAC, Tan et al. (2026) systematically summarized its outcomes, including the beneficial impact of EAC on both employee performance and innovation outcomes. In the context of EAC, AI undertakes routine cognitive tasks such as information searching, data processing, and pattern recognition, which directly reduces employees’ cognitive job demands. This allows employees to devote more energy, time, and expertise to EIB that require in-depth exploration and novel idea generation (Jia et al., 2024; Wilson & Daugherty, 2018).

Collaboration with AI provides reliable algorithmic support for employees’ creative attempts and reduces perceived risk. Through continuous monitoring and optimization of processes and outcomes, AI enhances employees’ confidence and security when exploring unfamiliar solutions or adopting new methods (Cannavale et al., 2022; Gandía et al., 2025). Such collaboration reduces the inherent uncertainty and failure costs in the innovation process, encourages employees to experiment and implement novel ideas, facilitates the translation of new ideas into actionable initiatives, and ultimately promotes the implementation of innovation outcomes (Lehmann et al., 2025). By interacting with AI, employees can learn and reshape their work knowledge and experience (Alavi et al., 2024), which is widely recognized as a critical catalyst for creativity (Wang & Peng, 2022; Y. Li et al., 2024).

**H1.**  *EAC is positively related to EIB.*

### 2.3. EAC and FWS

According to SCT, the environment is a key determinant that shapes individuals’ cognitive structures (Bandura, 1986). We argue that in the context of EAC, AI helps employees complete repetitive, monotonous, and non-innovative tasks, thereby improving work efficiency (Tang et al., 2022) and saving employees’ time and psychological resources. The saved time and resources allow employees to focus on self-development and future-oriented thinking, leading them to form clearer plans and goals, as well as a more explicit cognition of their desired future work state.

First, EAC frees up cognitive resources. Under traditional work models, employees are often trapped in repetitive, low-value routine tasks, leading to cognitive overload and difficulty thinking about long-term career development. Following the introduction of human–AI collaboration, intelligent algorithms can undertake cumbersome data processing and routine decision-making tasks (Tang et al., 2022; Tan et al., 2026). This task substitution effect liberates employees’ valuable attentional resources, enabling them to reallocate cognitive bandwidth to in-depth reflection and planning for their careers, thereby forming a clearer career vision for the future.

Second, the context of EAC enhances perceived control and reduces environmental uncertainty. Clear self-cognition is often based on environmental predictability. The powerful data analysis and forecasting capabilities of artificial intelligence provide employees with a stronger sense of job control and mastery experience (Parker & Grote, 2022). This elevated self-efficacy assures employees that they can cope with future challenges, thereby facilitating the construction of a more concrete and clear future work self-image. In summary, we propose:

**H2.**  *EAC is positively related to employees’ FWS.*

### 2.4. The Mediating Role of FWS

FWS refers to the clarity with which individuals represent their desired future work states or occupational roles (Zhang et al., 2017; Chang et al., 2026). SCT emphasizes that forethought is a core feature of human cognition (Bandura, 1986); individuals guide current behavior by setting goals and anticipating future states. The self-regulation mechanism of SCT suggests that humans are not passively shaped by the environment and internal drives alone, but can proactively regulate their thoughts, emotions, and behaviors. EIB is generally defined as proactive behavior that generates novel and valuable outcomes. We propose that employees’ clear vision of their future work serves as a key intrinsic motivation driving EIB.

According to the goal-directed mechanism of SCT, a highly clear future work self represents a strong form of intrinsic motivation (Zhang et al., 2017; Chang et al., 2026). First, a strong future work self provides employees with clear directional guidance. When employees form a clear cognitive representation of “who they will become in the future,” this vision acts as a behavioral guide (Strauss et al., 2012). Although innovation involves risk and uncertainty, it also serves as a critical path to achieving career advancement and moving toward the ideal future self. To approach their ideal self, employees are more willing to proactively explore new methods and technologies, demonstrating capabilities consistent with their future roles.

Second, a clear future work self stimulates discrepancy-reduction motivation. A distinct future vision highlights the gap between the ideal future self and the current reality (Chang et al., 2026), generating constructive psychological tension (Oh, 2020). To alleviate this tension, employees are unwilling to remain complacent with the status quo. Instead, they tend to adopt change-oriented behavior—namely, EIB—by breaking routines and improving job performance, thereby accelerating the achievement of their future work self. Beyond these motivational mechanisms, FWS offers a distinct pathway. Unlike self-efficacy or work engagement, FWS is inherently future-oriented and identity-based—it captures employees’ cognitive representation of their desired career future (Strauss et al., 2012). This future-oriented, self-definitional nature makes FWS particularly relevant in the EAC context, where AI exposure may prompt employees to reassess their long-term career trajectories (Voigt & Strauss, 2024). Thus, FWS provides a theoretically grounded mechanism that complements existing motivational accounts rather than duplicating them. In summary, we propose:

**H3.**  *FWS serves as a mediator in the relationship between EAC and EIB.*

### 2.5. The Moderating Role of CSE

Although the human–AI collaboration context can shape employees’ cognition, SCT states that the influence of the external environment on individual cognition is constrained by personal traits (Bandura, 1986). This study introduces CSE as a moderating variable. CSE reflects individuals’ fundamental appraisals of their own ability, worth, and capacity to control the environment (Srivastava et al., 2010). Drawing on the resource complementarity logic derived from complementarity theory, we propose that external resources (EAC) and internal psychological resources (CSE) may function in a substitutive rather than purely additive manner in shaping FWS. Specifically, this logic predicts that when internal resources are scarce (i.e., low CSE), individuals should exhibit greater sensitivity to external inputs, such that the positive effect of EAC on FWS becomes more pronounced; conversely, when internal resources are abundant (i.e., high CSE), the marginal cognitive benefit of EAC is expected to be attenuated. This pattern is consistent with a resource-substitution interpretation, wherein the task assistance and informational support provided by EAC may have a stronger cognitive impact precisely for those who lack sufficient internal self-evaluative resources. We use the term “compensatory” cautiously, as a descriptive label for this moderation pattern, while acknowledging that the psychological process of compensation was not directly measured in this study.

Conversely, employees with high CSE already possess high self-efficacy and emotional stability, and tend to take initiative in managing their careers. Their baseline FWS is inherently high, leaving less room for EAC to provide incremental gains. This pattern, stronger effects for low CSE and weaker effects for high CSE, reflects the same resource-substitution mechanism: external resources matter most when corresponding internal resources are deficient, and their marginal benefit diminishes as internal resources become sufficient. In summary, we propose:

**H4.**  *CSE moderates the positive relationship between EAC and FWS. That is, the promoting effect of human–AI collaboration on FWS is stronger when CSE is low, whereas this effect is weaker or even disappears when CSE is high.*

### 2.6. Moderated Mediation Effect

The indirect mechanism through which EAC promotes innovation behavior by enhancing FWS is bounded by individual CSE. For employees with low CSE, the resource compensation effect of EAC is maximized, which effectively activates the clear construction of their FWS and further releases strong intrinsic motivation to engage in EIB. For employees with high CSE, however, due to the self-sufficiency of internal resources, the indirect effect of EAC on innovation behavior via the cognitive path of FWS is relatively weak. Accordingly, we hypothesize:

**H1.**  *CSE moderates the mediating relationship of FWS between EAC and EIB. That is, the lower CSE is, the stronger the mediating effect; the higher CSE is, the weaker or even nonsignificant the mediating effect becomes.*

**Figure 1:**
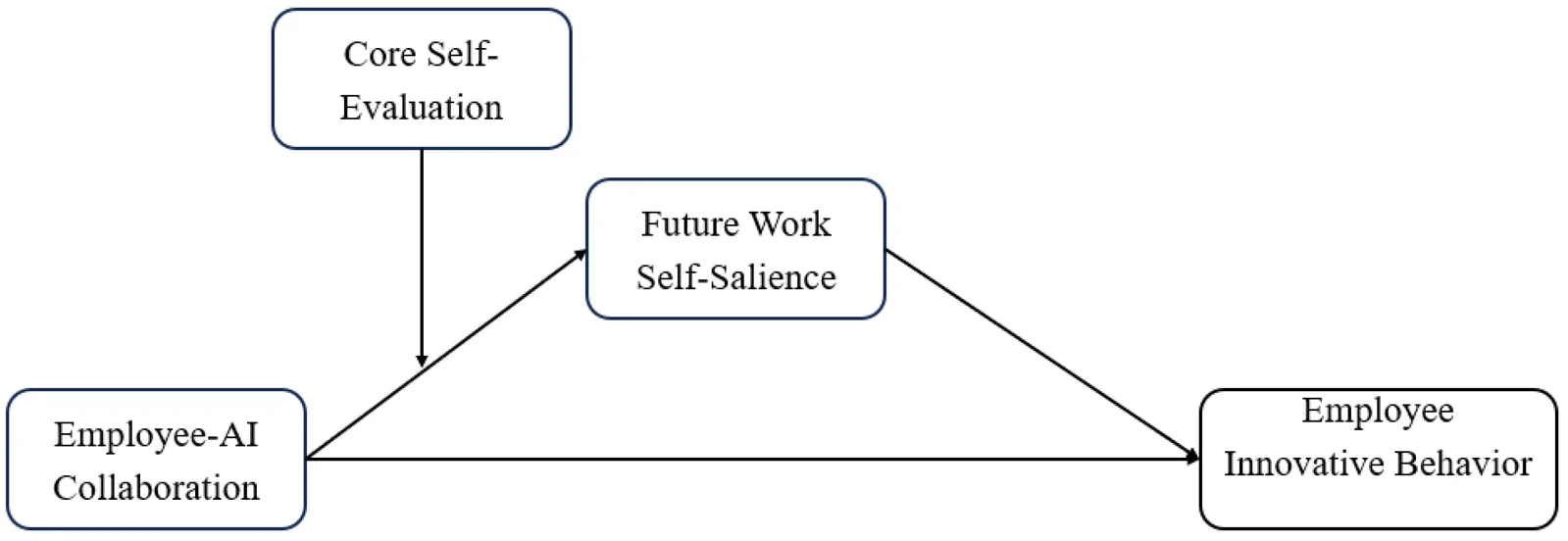
Theoretical model.

The theoretical model is depicted in Figure 1.

## 3. Method

### 3.1. Sample and Data Collection

Data were collected via three-wave questionnaire surveys among full-time employees through the Credamo platform from August 2025 to November 2025. Before the formal investigation, the research team assured the participants of anonymous processing and strict confidentiality of personal information, in order to alleviate their concerns and guarantee the authenticity of the responses. Over the course of data collection, the questionnaire clearly stated the academic purposes and emphasized that there were no “right” or “wrong” answers. At the same time, the academic terms such as “stakeholders” were defined contextually (for example, defined as “customers and suppliers with business relations to the company”), in order to avoid response deviations caused by semantic misunderstandings.

To alleviate the common method bias, the following strategies were adopted in this study: trust was established through an anonymous confidentiality mechanism; and the questions of the EAC, FWS, and EIB were distributed across different questionnaire modules to avoid dimension concentration. We also provided operational definitions for key constructs, and adopted a three-wave time-lagged research design. In particular, at Time 1, we collected demographic information, EAC, and CSE. FWS was measured at Time 2, roughly two weeks after the first survey. After another two-week interval, at Time 3, we assessed EIB. During data cleaning, we excluded responses that met any of the following criteria: more than 30% missing data for any single variable, more than 20% missing data across all items, responses with logical inconsistencies, or those containing 10 or more consecutive identical answers. This procedure resulted in a final valid sample of 457 participants, representing a valid response rate of 88.57%. We recruited full-time employees using convenience (nonprobability) sampling. While this approach may modestly restrict the generalizability of our findings, it enabled feasible and efficient data collection under real-world field conditions.

The final sample comprised 457 full-time employees. Of the 516 respondents at T1, 489 completed the T2 survey and 457 completed all three waves, representing an overall retention rate of 88.57%. Responses were matched using unique participant codes generated by the Credamo platform. No incentives were offered. Eligibility required full-time employment with at least six months of work experience and regular use of AI tools in daily tasks. The sample covered 28 provinces in China, primarily Anhui, Guangdong, and Jiangsu. The valid response rate of 88.57% was calculated as the proportion of T1 respondents who completed all three waves (457/516). Female respondents accounted for 61.5% of the sample. Regarding age distribution, those aged between 21 and 30 accounted for 38.1%, and those aged between 31 and 40 accounted for 52.1%. In terms of educational background, those with a college degree or below accounted for 9.8%, those with a bachelor’s degree accounted for 70.5%, and those with a postgraduate degree or above accounted for 19.7%. Regarding work experience, 27.8% of participants had less than 5 years of experience, 46.0% had 6–10 years, 18.2% had 11–15 years, and 8.1% had 16 years or more. Regarding industry distribution, 27.1% of participants worked in manufacturing, and 33.0% worked in the internet industry.

### 3.2. Measures of Variables

The original English-language scales were translated into Chinese using a standard translation and back-translation procedure, with appropriate adjustments made to the expressions according to the research context (Brislin, 1970). All key variables were rated on a 5-point Likert scale (1 = strongly disagree, 5 = strongly agree).

EAC (α = 0.745) was assessed using the 5-item scale developed by Kong et al. (2023) to measure employee–AI collaboration. A sample item is “Artificial intelligence participates in my decision-making process.”

CSE (α = 0.843) was measured using a 12-item scale developed by Judge et al. (2003). A representative item is “I am confident I will get the success I deserve.”

FWS (α = 0.764) was measured using a 5-item scale developed by Strauss et al. (2012). A representative item is “I can easily picture my future work self.”

EIB (α = 0.816) was evaluated using a 6-item scale originally developed by Scott and Bruce (1994). A representative item is “I generate ideas for new technologies, processes, or products.”

Control variables. We controlled for gender (0 = male, 1 = female), age (years), education, work experience (years), position (1 = entry-level to 5 = senior management), and functional section (categorical). Position and section were included as they may influence access to AI tools and opportunities for innovation (Jia et al., 2024).

## 4. Results

### 4.1. Confirmatory Factor Analysis

To examine the discriminant validity of the key variables, CFA was conducted. The result showed that the four-factor model including EAC, FWS, CSE, and EIB exhibited a satisfactory model fit (*χ*^2^/*df* = 2.080, RMSEA = 0.049, CFI = 0.918, IFI = 0.919, and SRMR = 0.046). The model fit indices for all alternative models are presented in Table 1.

### 4.2. Common Method Bias

We used a multiwave design to reduce the risk of common method bias. First, Harman’s single-factor test (Podsakoff et al., 2003) was performed on all indicators of the four key variables, namely EAC, FWS, CSE, and EIB. The results indicated that multiple factors emerged, with the first factor explaining only 30.71% of the total variance—below the conventional threshold for common method bias. Furthermore, CFA with an added common method factor was conducted by incorporating a latent method factor into the four-factor model (Gu & Wen, 2017). The findings indicated no significant improvement in model fit: changes in RMSEA and SRMR were less than 0.05, while changes in CFI and IFI were less than 0.01, suggesting that common method bias was unlikely to pose a serious concern in this study. Therefore, as no significant common method bias was detected in the data, subsequent statistical tests could be conducted.

### 4.3. Correlation Analysis

Table 2 displays descriptive statistics and correlations for all variables. EIB was significantly positively correlated with FWS, CSE, and EAC; and EAC was also significantly and positively correlated with FWS. These correlations provided preliminary support for the proposed relationships, which were examined further in the hypothesis tests.

### 4.4. Hypothesis Testing

All analyses were conducted using SPSS 26.0 and PROCESS macro (Model 7). Variables were mean-centered prior to creating interaction terms. Bootstrap tests used 5000 resamples to construct 95% bias-corrected confidence intervals. Simple slopes were examined at one standard deviation above and below the mean of CSE to define “high” and “low” groups. Table 3 presents the results of hierarchical regression analysis. The maximum variance inflation factor (VIF) observed was 2.92, which is below the threshold of 5, indicating no significant multicollinearity in the regression models.

**Figure 2:**
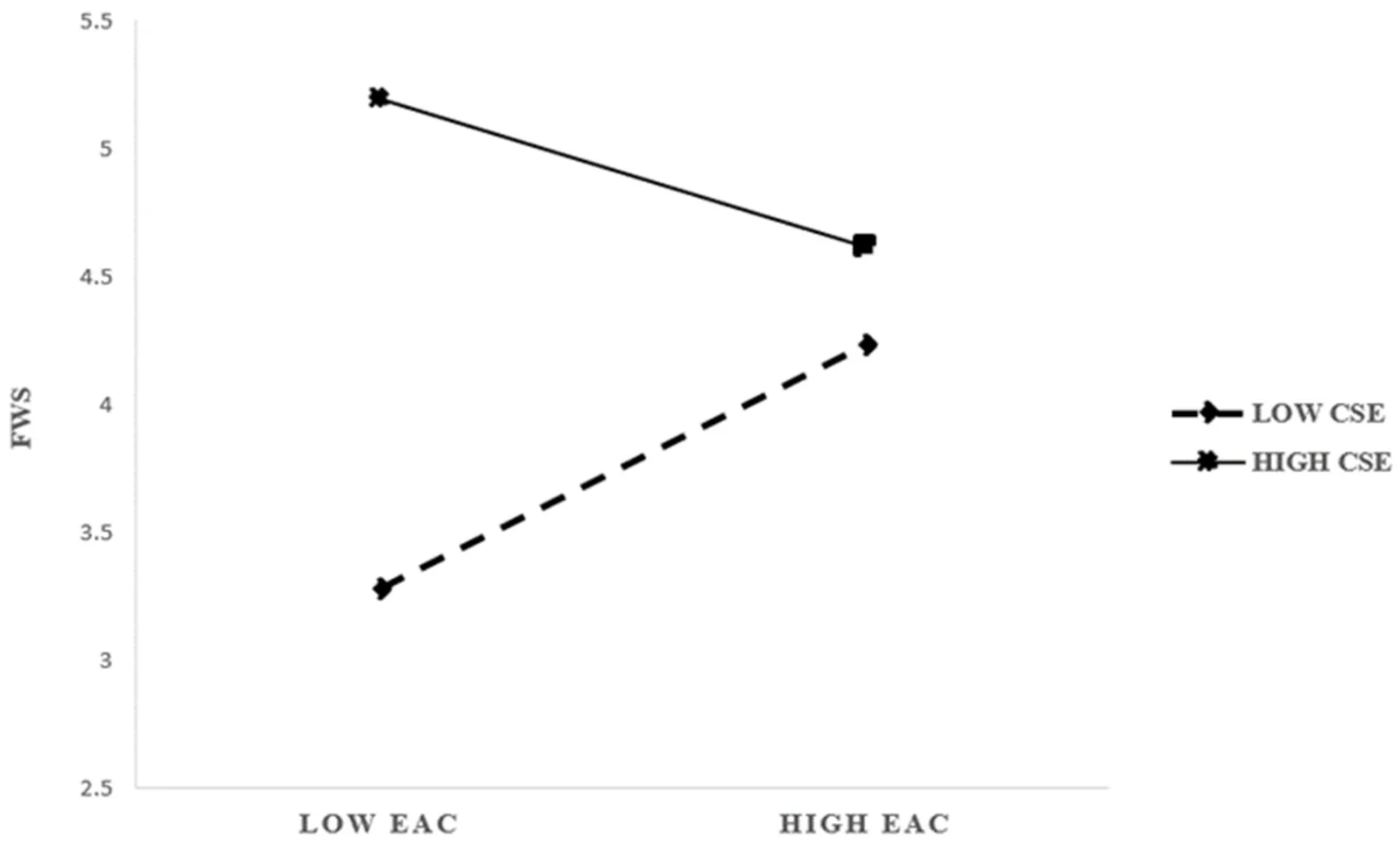
Moderating role of CSE.

Hypothesis 1 proposed that EAC positively affects EIB. As shown in Model 6 of Table 3, EAC significantly and positively predicted innovation behavior (b = 0.389, *p* < 0.001), thus supporting H1. Hypothesis 2 proposed that EAC positively affects FWS. Model 2 of Table 3 showed that EAC significantly and positively predicted future work self-salience (b = 0.466, *p* < 0.001), thus supporting H2. Further analysis revealed that after adding FWS (Model 7), the coefficient of EAC decreased to 0.247 (significant), suggesting that FWS mediates the relationship between EAC and EIB. In addition, the bootstrap analysis showed that the mediating effect was 0.165, with a 95% bootstrap confidence interval of [0.068, 0.280], which did not include zero (see Table 4). The total effect reported in Table 4 (0.451) and the EAC coefficient in Table 3 Model 6 (0.389) represent the same overall effect estimated using different methods (bootstrapping vs. hierarchical regression); the slight numerical difference is attributable to methodological variation rather than substantive inconsistency. Critically, the decomposition in Table 4 shows that this total effect is partitioned into a direct effect (0.286) and an indirect effect via FWS (0.165), supporting the hypothesized mediation pathway. These results indicated that FWS significantly mediated the relationship between EAC and EIB, supporting H3. Model 4 shows that the interaction term between EAC and CSE had a significant predictive effect on FWS (b = −0.206, *p* < 0.001). Simple slope analysis (see Figure 2) revealed that EAC had a significant positive effect on FWS at low levels of CSE, whereas the effect of EAC on FWS was not significant at high levels of CSE. This suggests that CSE conditions the relationship between EAC and future work self-salience, supporting H4. As shown in Table 5, the conditional indirect effect of EAC on EIB via FWS was significant for employees with low CSE (effect = 0.091, SE = 0.040, 95% BootCI [0.028, 0.186]), but nonsignificant for those with high CSE (effect = −0.020, SE = 0.034, 95% BootCI [−0.100, 0.038]). The index of moderated mediation was −0.111, with a 95% BootCI [−0.243, −0.025] excluding zero, supporting the hypothesized moderated mediation effect.

## 5. Discussion

Drawing on SCT and complementarity theory, this study develops and validates a moderated mediation model examining how and when EAC promotes EIB. The empirical results yield three key findings: EAC has a significant positive predictive relationship with EIB; FWS serves as a mediating pathway linking EAC to EIB; and CSE moderates this mediated relationship, such that the indirect effect is stronger among low-CSE employees. These findings offer two important insights. First, they confirm that EAC serves as an effective environmental stimulus that enhances employees’ future-oriented self-concept, which in turn drives EIB. Second, and more importantly, they reveal that the cognitive benefits of EAC are not universally distributed but are contingent upon individual CSE—a finding that diverges from the conventional “more resources are better” assumption.

### 5.1. Theoretical Implications

First, this study extends and refines the application of SCT to the field of human–AI collaboration. Existing scholars have adopted various theories in research on human–AI collaboration. For instance, Y. Li et al. (2024) applied signaling theory to demonstrate that human–AI collaboration can improve people’s acceptance of chatbots; Wu et al. (2025) used self-determination theory to verify that human–AI collaboration enhances employee work engagement. Although these studies from diverse perspectives have greatly contributed to the literature on human–AI collaboration, few studies have approached this topic from the lens of SCT. More importantly, prior SCT-based research has predominantly emphasized a synergistic logic—that favorable environments and strong personal attributes combine to amplify desired outcomes (Bandura, 1986, 1991). Our findings reveal a moderation pattern that is theoretically consistent with a resource-substitution logic: EAC significantly predicts FWS only among low-CSE employees, whereas this effect becomes nonsignificant among their high-CSE counterparts. This observed pattern, while not a direct test of the compensatory process itself, is consistent with a resource-substitution interpretation. It thus lends indirect empirical support to the complementarity-based reasoning that external resources become more influential when internal resources are deficient. Beyond identifying this observed resource-substitution pattern, our findings extend SCT research by identifying CSE as a boundary condition that specifies when environmental resources (EAC) become more influential for FWS—namely, when corresponding internal resources are low. This refines SCT’s person–environment interaction principle by demonstrating that external and internal resources can operate in a substitutive rather than merely additive manner. Importantly, this extension is situated in the under-explored context of human–AI collaboration, where SCT has rarely been applied.

Second, this study introduces FWS as a cognitive mediator, identifying a concrete mechanism through which EAC fosters innovation. Existing studies on EAC have examined mediators such as basic quality of work-life needs, intrinsic motivation, and work engagement (Wu et al., 2025; Y. Liu & Li, 2025), yet to our knowledge, no research has adopted FWS as a mediator. While prior research on AI and innovation has predominantly focused on motivational pathways, our findings demonstrate that FWS—operating through long-term goal orientation and discrepancy-reduction motivation—represents a conceptually distinct pathway. This discovery not only enriches research on the antecedents of FWS but also illuminates a specific cognitive pathway through which EAC shapes employee innovation, confirming that future-oriented cognition serves as a critical bridge connecting intelligent technology and innovation performance.

Third, this study introduces CSE as a moderator and clarifies the boundary conditions under which EAC functions from the perspective of resource complementarity. Previous studies found that CSE is a predictor of academic burnout, with a dynamic circular relationship between them (J. Li et al., 2024); CSE can also influence prosocial behavior, with reciprocal effects over time (Xia et al., 2026). However, most of these studies treat CSE as an antecedent or an outcome variable, with relatively few studies regarding it as a boundary condition. More importantly, prior CSE research has predominantly adopted an amplification perspective, suggesting that high-CSE individuals capitalize more effectively on favorable conditions. Our counterintuitive finding—that EAC’s indirect effect on EIB via FWS is stronger for low-CSE employees—challenges this assumption, reframing CSE from a universal “resource multiplier” to a context-dependent “resource substitute.”

### 5.2. Practical Implications

First, organizations may consider actively promoting the human–AI collaboration model as a new engine to stimulate employee innovation. The findings indicate that EAC is significantly and positively associated with EIB. This suggests that when introducing AI, organizations should not regard AI merely as an automated alternative tool for cost reduction and efficiency improvement, but position it as a partner that empowers employees. Managers may wish to encourage employees to interact deeply with AI systems in daily work. By providing targeted AI skills training (e.g., prompt engineering, human–AI interaction logic), building a tolerant culture for AI trial and error, and offering time and resource support for solving complex problems through EAC, organizations may be able to foster the stimulation of employees’ innovative potential.

Second, organizations could focus on the reconstruction of employees’ career cognition and consider using AI to assist in building FWS. This study finds that FWS plays a critical bridging role between EAC and EIB. Amid the career uncertainty brought by the rapid popularization of AI, employees are prone to confusion. Therefore, when implementing AI tools, managers could accompany them with career intervention measures. On the one hand, organizations could clearly depict the “future job landscape empowered by AI” for employees, helping them recognize that after AI takes over repetitive tasks, their core value may shift toward creativity and strategic thinking. On the other hand, organizations could establish “model employees of human–AI collaboration” to provide role models for other employees to observe and learn from, helping them clarify and establish their ideal future career state with the help of AI, which may stimulate their intrinsic motivation for proactive innovation.

Finally, organizations should implement differentiated AI empowerment strategies and focus on providing compensatory resources to employees with low CSE. This is the most distinctive and practically valuable finding of this study. The positive effect of EAC on future work self-salience depends on employees’ CSE. This effect is significant only among employees with low CSE, indicating a resource-compensatory pattern that addresses deficits rather than merely amplifying existing strengths. The findings tentatively imply the following: For employees with low CSE (e.g., low self-confidence, confusion about career development): managers may consider using AI as a cognitive support tool and prioritize guiding these employees to use it. The abundant information, immediate feedback, and low-risk experimentation opportunities provided by AI may help them build workplace confidence and clarify their career paths. For high-performing employees with high CSE: since they may already possess relatively clear self-cognition and career plans, the incremental benefit of EAC on FWS appears limited—a pattern that could be interpreted as reflecting a ceiling effect—and managers do not necessarily need to push them to “find future direction” through AI. Instead, they may be allowed to use AI as a tool to improve efficiency at their own pace. Organizations should avoid a “one-size-fits-all” promotion model and strive for precision management in AI empowerment.

### 5.3. Limitations and Future Outlook

First, this study examined EAC as an overall construct without distinguishing between different types of artificial intelligence technologies. As technology has advanced, the manifestations of AI have grown progressively more varied. Generative AI (such as ChatGPT, which emphasizes content creation and interaction) and decision-support AI (tools that emphasize data analysis and algorithmic recommendations) may elicit different psychological and cognitive processes when employees interact with them. Future research could focus on specific types of AI (especially generative AI), develop more targeted measurement scales, and explore the differentiated impacts of different types of EAC on EIB.

Second, based on complementarity theory, this study mainly examines the moderating role of individual traits (CSE). However, employees’ cognitive restructuring and EIB are also deeply embedded in the organizational context. Future research could incorporate organizational-level environmental variables into the model, such as exploring the cross-level moderating effects of organizational innovation climate, inclusive leadership, or AI application climate in human–AI collaboration. In addition, the introduction of AI may represent a double-edged sword. Future studies could consider incorporating negative pathways (e.g., AI anxiety, job insecurity, or perceptions of dehumanization) to construct a dual-pathway model that includes both positive empowerment (e.g., social cognition) and negative depletion. This would offer a fuller, more balanced view of EAC’s complex effects. Additionally, although we used the established EAC scale developed by Kong et al. (2023), its items focus primarily on behavioral reliance on AI, which may not fully capture the bidirectional and interactive nature of collaboration. Future research would benefit from developing a more comprehensive measure that more precisely reflects the full scope of EAC.

Third, despite the three-wave time-lagged design, all focal variables were measured via employee self-reports. This design does not establish causal relationships among EAC, FWS, and EIB, and remains vulnerable to same-source bias. For instance, employees who hold positive work perceptions may rate all constructs more favorably, potentially inflating the observed associations. Future research should incorporate supervisor-rated or objective measures of EIB, or adopt experimental designs, to strengthen causal inference.

Fourth, although our analyses relied primarily on hierarchical regression with the PROCESS macro, future research could employ structural equation modeling (SEM) for a more comprehensive assessment of the underlying process. Additionally, the measurement of EAC warrants particular caution. The adopted scale may reflect AI use or dependence more than genuinely reciprocal human–AI collaboration, which may constrain its construct validity. Future research would benefit from developing more comprehensive measures that capture mutual adaptation and shared decision-making between employees and AI systems.

## 6. Conclusions

This empirical study examines the association between EAC and EIB. EAC exhibits a significant positive predictive effect on EIB, a relationship that is mediated by FWS. The relationship between EAC and FWS is contingent upon CSE, identifying the boundary condition under which EAC functions. Consistent with a resource complementarity perspective, our findings suggest a pattern that is interpretable as compensatory: For employees with low CSE, who tend to possess fewer internal psychological resources, the task assistance and informational support from EAC may offset this deficit. EAC was more strongly associated with FWS among employees with low CSE. For employees with high CSE, who already possess strong self-efficacy and environmental control and may already have relatively high levels of FWS, the marginal gain of EAC is relatively limited, resulting in a weaker effect on FWS.

## Figures and Tables

**Table 1:** Comparison of measurement models.

Model	*χ* ^2^	*df*	*χ*^2^/*df*	CFI	IFI	TLI	RMSEA	SRMR
Four-Factor Model (*EAC*, *FWS*, *CSE*, *EIB*)	642.851	309	2.080	0.918	0.919	0.907	0.049	0.046
Three-Factor Model (EAC *+ FWS*, *CSE*, *EIB*)	1016.275	321	3.166	0.829	0.830	0.813	0.069	0.058
Two-Factor Model (EAC *+ FWS + CSE*, *EIB*)	1135.330	323	3.515	0.800	0.801	0.783	0.074	0.061
One-Factor Model (EAC *+ FWS + CSE + EIB*)	1423.237	324	4.393	0.729	0.731	0.707	0.086	0.071

Note: EAC = employee–AI collaboration, FWS = future work self-salience, CSE = core self-evaluation, EIB = employee innovative behavior.

**Table 2:** Means, standard deviations, and correlation coefficients of variables (N = 457).

	1	2	3	4	5	6	7	8	9	10
1. gender	1									
2. age	0.021	1								
3. edu	0.019	0.015	1							
4. work	−0.022	0.792 **	−0.087	1						
5. position	0.048	0.390 **	0.350 **	0.343 **	1					
6. section	0.081	−0.044	−0.179 **	0.041	−0.012	1				
7. EAC	0.014	0.076	0.178 **	−0.040	0.160 **	−0.184 **	1			
8. CSE	−0.032	0.159 **	0.230 **	0.075	0.277 **	−0.234 **	0.486 **	1		
9. FWS	0.017	0.080	0.181 **	−0.006	0.208 **	−0.057	0.493 **	0.667 **	1	
10. EIB	−0.064	0.105 *	0.153 **	0.021	0.242 **	−0.167 **	0.439 **	0.549 **	0.462 **	1
mean	1.610	2.750	2.140	2.110	2.000	3.180	4.223	4.132	4.289	4.252
SD	0.487	0.726	0.567	1.007	0.970	2.135	0.462	0.480	0.499	0.536

Note: * *p* < 0.05, ** *p* < 0.01; EAC = employee–AI collaboration, FWS = future work self-salience, CSE = core self-evaluation, EIB = employee innovative behavior.

**Table 3:** Regression analysis results for hypothesis testing.

Variables	FWS				EIB		
	Model 1	Model 2	Model 3	Model 4	Model 5	Model 6	Model 7
gender	0.001	−0.003	0.019	0.016	−0.070	−0.074	−0.073
age	0.147	0.054	0.023	0.029	0.133	0.056	0.039
edu	0.098	0.059	0.016	0.014	0.031	−0.001	−0.019
work	−0.173 *	−0.067	−0.069	−0.05	−0.158 *	−0.069	−0.049
position	0.175 **	0.115 *	0.021	0.004	0.236 ***	0.185 ***	0.15 **
section	−0.024	0.046	0.125 ***	0.112 **	−0.141 **	−0.083	−0.097 *
EAC		0.466 ***	0.223 ***	0.1 *		0.389 ***	0.247 ***
FWS							0.305 ***
CSE			0.581 ***	0.571 ***			
EAC × CSE				−0.206 ***			
R^2^	0.068	0.266	0.501	0.526	0.100	0.238	0.306
DR^2^	0.068	0.198	0.234	0.025	0.100	0.138	0.068
F	5.510	23.304	56.148	55.060	8.378	20.054	24.729

Note: * *p* < 0.05, ** *p* < 0.01, *** *p* < 0.001.

**Table 4:** Decomposition of total effects.

EAC → FWS → EIB				
Effect Type	Effect	SE	95% CI	
Total Effect	0.451	0.050	0.353	0.549
Direct Effect	0.286	0.054	0.180	0.392
Indirect Effect	0.165	0.054	0.068	0.280

**Table 5:** Test of moderated mediation effect.

	Mediation Effect Estimate	SE	95% CI	
low group	0.091	0.040	0.028	0.186
high group	−0.020	0.034	−0.100	0.038
index of moderated mediation	−0.111	0.056	−0.243	−0.025

## Data Availability

Study data are available upon reasonable request.
